# Differential expression of CD300a/c on human T_H_1 and T_H_17 cells

**DOI:** 10.1186/1471-2172-12-62

**Published:** 2011-11-02

**Authors:** Venkateswara R Simhadri, John L Mariano, Qing Zhou, Karen E DeBell, Francisco Borrego

**Affiliations:** 1 Laboratory of Molecular and Developmental Immunology, Division of Monoclonal Antibodies, Office of Biotechnology Products, Center for Drug Evaluation and Research, Food and Drug Administration, Bethesda, Maryland, USA

## Abstract

**Background:**

Human memory CD4^+ ^T cells can be either CD300a/c^+ ^or CD300a/c^- ^and subsequent analyses showed that CD4^+ ^effector memory T (T_EM_) cells are mostly CD300a/c^+^, whereas CD4^+ ^central memory T (T_CM_) cells have similar frequencies of CD300a/c^+ ^and CD300a/c^- ^cells.

**Results:**

Extensive phenotypical and functional characterization showed that in both T_CM _and T_EM _cells, the CD300a/c^+ ^subset contained a higher number of T_H_1 (IFN-γ producing) cells. Alternatively, T_H_17 (IL-17a producing) cells tend to be CD300a/c^-^, especially in the T_EM _subset. Further characterization of the IL-17a^+ ^cells showed that cells that produce only this cytokine are mostly CD300a/c^-^, while cells that produce IL-17a in combination with other cytokines, especially IFN-γ, are mostly CD300a/c^+^, indicating that the expression of this receptor is associated with cells that produce IFN-γ. Co-ligation of the TCR and CD300a/c in CD4^+ ^T cells inhibited Ca^2+ ^mobilization evoked by TCR ligation alone and modulated IFN-γ production on T_H_1 polarized cells.

**Conclusion:**

We conclude that the CD300a/c receptors are differentially expressed on human T_H_1 and T_H_17 cells and that their ligation is capable of modulating TCR mediated signals.

## Background

Upon encounter with the antigen in secondary lymphoid tissues, naïve CD4^+ ^T cells initiate a vigorous clonal expansion. This expansion leads to the differentiation and specialization into functionally distinct T helper (T_H_) cell subsets or lineages. Each T_H _subset is involved in tailoring immune responses specific to a wide range of antigens. They are characterized by the expression of specific cell surface receptors, and distinct transcription factors that lead to the secretion of a particular set of cytokines [1]. For example, T_H_1 cells express the transcription factor T-bet and secrete IFN-γ, TNF-α and IL-2. They also express the chemokine receptors CCR5 and CXCR3 and the cytokine receptors IL-12Rβ2 and IL-18Rα. T_H_1 cells play an important role in the resistance against intracellular pathogens and in the pathogenesis and maintenance of certain autoimmune diseases [2-13]. Another T_H _subset, T_H_17 cells, express the transcription factor RORγt, secrete IL-17a, IL-17f and IL-22 and are characterized by the expression of the chemokine receptor CCR6, the cytokine receptors IL-23R and IL-1R1 and the C-type lectin receptor CD161. T_H_17 cells play a very important role in the defense against extracellular pathogens and in the pathogenesis of autoimmune diseases [14-22]. Other T_H _subsets include T_H_2, T follicular helper (T_FH_) and induced regulatory T (iTreg) cells [1,23]. The classical view of distinct and terminally differentiated lineages is currently challenged by many findings showing a degree of plasticity and flexibility in the T_H _subsets that can be represented as a series of transitions from less to more stable states [24-26]. Furthermore, there are many CD4^+ ^T cells that do not fit the profile of the subsets described above in that they produce cytokines ascribed to more than one lineage. For instance, some human IL-17a or IL-4 producing cells were found to also produce IFN-γ [14,27-29]. Additionally, T_H _subsets can also be subdivided according to the expression of cell surface receptors. We have previously reported that the expression of CD300a/c distinguishes a subset of T_H_1 cells that is more polyfunctional and, after stimulation, up-regulates the T-box transcription factor Eomesodemin [30].

CD300a is an immunomodulatory receptor that belongs to the CD300 family of activating/inhibitory receptors [31]. It is a type I transmembrane receptor that has three classical immunoreceptor tyrosine-based inhibitory motifs (ITIMs) and one non-classical ITIM in its cytoplasmic tail and a single V-like Ig extracellular domain. This receptor is expressed on cells of both myeloid and lymphoid lineages and the ligand is not known [31]. Several *in vitro *studies have shown that CD300a ligation is capable of inhibiting the eosinophil response to eotaxin and IL-5 [32], NK cell mediated cytotoxicity [33,34], B cell receptor (BCR) mediated Ca^2+ ^mobilization and NFAT translocation [35], FcεRI mediated activation of mast cells [36] and FcγRIIa mediated Ca^2+ ^flux and reactive oxygen species (ROS) production in neutrophils [37]. *In vivo *studies have also shown the inhibitory potential of CD300a. For instance, treatment of mice with a bispecific antibody linking CCR3 to CD300a reversed remodeling and airway inflammation in a model of asthma [38]. Furthermore, genetic studies have revealed that a non-synonymous mutation in the CD300a extracellular domain is linked to psoriasis susceptibility [39] and that is implicated in the development of Alzheimer's disease [40]. Other studies have shown that circulating CD4^+^CD45RO^+ ^T cells exhibit lower expression of CD300a/c in psoriasis patients compared with healthy donors [41]. The expression of CD300a/c is also down-regulated in B cells from HIV infected patients, suggesting that this receptor may contribute to the B cell dysfunction observed in HIV induced immunodeficiency [35]. CD300a, along with three other genes, has been identified as a blood biomarker that can be used for the differentiation of patients with ulcerative colitis from patients with Crohn's disease and noninflammatory diarrhea [42].

It has been previously shown by real time PCR that both receptors, CD300a and CD300c, are expressed on human CD4^+ ^T cells [41]. However, with the available monoclonal antibodies it is impossible to distinguish CD300a from CD300c on the cell surface [34]. The extracellular domains of CD300a and CD300c are highly homologous, showing 80% identity [31,34]. While the transmembrane region of CD300c contains a charged amino acid that is important for its association with the immune receptor tyrosine-based activation motif (ITAM) bearing FcεRγ chain, and therefore delivers activating signals [43], CD300a, as mentioned above, transmits inhibitory signals through the ITIMs containing intracellular tail [34].

In this report, using the anti-CD300a/c E59.126 mAb, we show that CD300a/c is preferentially expressed on CD4^+ ^effector memory T (T_EM_) cells. We show that CD4^+ ^T cells producing only IL-17a are mostly CD300a/c^- ^while CD4^+ ^cells producing IL-17a in combination with other cytokines, such as IFN-γ, TNF-α and/or IL-2 are mostly CD300a/c^+^. Furthermore, we investigate the ability of CD300a/c receptors to modulate TCR mediated Ca^2+ ^flux and IFN-γ production of T_H_1 polarized cells.

## Results

### CD300a/c is highly expressed on T_EM _CD4^+ ^T cells

A recent manuscript has reported that CD300a and CD300c are impossible to distinguish on the cell surface, using a panel of mAbs [34]. Therefore, we first tested the binding specificity of the E59.126 mAb, an antibody previously described as CD300a specific [30,32,33,35-37,44]. Our results showed that this antibody recognizes both CD300a and CD300c receptors on transiently transfected 293T cells (data not shown). Consequently, hereafter we will refer as CD300a/c^+ ^to cells that specifically bind the E59.126 mAb.

**Figure 1:**
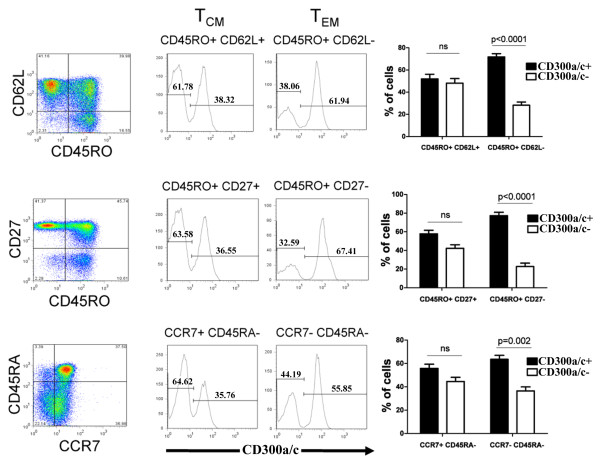
**Flow cytometric analysis of human peripheral blood CD4^+ ^T cells**. The expression of CD300a/c on central (T_CM_) and effector memory (T_EM_) CD4^+ ^T cells was determined with three staining strategies: CD45RO and CD62L (upper panels), CD45RO and CD27 (middle panels) and CD45RA and CCR7 (lower panels). A representative donor is shown for each staining. Bar graphs represent the average ± SEM of the percentage of CD300a/c^+ ^cells within T_CM _and T_EM_. Results shown are from 12 healthy donors.

We have previously published that naïve CD4^+ ^T cells express low amounts of CD300a/c, whereas memory CD4^+ ^T cells can be subdivided into CD300a/c^+ ^and CD300a/c^- ^populations [30]. However, it is not known if within the memory CD4^+ ^T cells there are differences in the expression of CD300a/c between the CD4^+ ^effector memory (T_EM_) and central memory (T_CM_) cells. We used three staining strategies to delineate the expression of CD300a/c by T_EM _and T_CM _CD4^+ ^T cells. Each one of these strategies is based on the combination of two markers: CD45RA and CCR7 [45], CD45RO and CD62L [46], or CD45RO and CD27 [45,47]. Our results show that T_EM _cells, characterized by the phenotype CD45RA^-^CCR7^-^, CD45RO^+^CD62L^- ^or CD45RO^+^CD27^-^, are predominantly CD300a/c^+ ^(~70%), whereas T_CM _cells, defined by the phenotype CD45RA^-^CCR7^+^, CD45RO^+^CD62L^+ ^or CD45RO^+^CD27^+^, have similar percentage of CD300a/c^+ ^and CD300a/c^- ^cells (Figure 1).

### CD300a/c expression in cytokine producing T_CM _and T_EM _CD4^+ ^T cells

**Figure 2:**
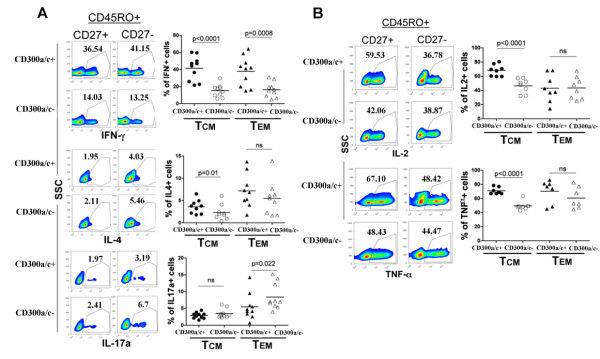
**Flow cytometric analyses of stimulated CD4^+ ^T cells**. Purified CD4^+ ^T cells were stimulated with PMA and ionomycin for 4-5 h in the presence of GolgiStop (monensin). The expression of CD300a/c was determined in the T_CM _(CD45RO^+^CD27^+^) and T_EM _(CD45RO^+^CD27^-^) cytokine producing cells. A representative healthy donor is shown for (A) the IFN-γ, IL-4 and IL-17a, and (B) IL-2 and TNF-α producing cells. Each graph represents the percentage of CD300a/c^+ ^and CD300a/c^- ^T_CM _and T_EM _cells that produce a given cytokine. Each symbol corresponds to a different donor.

Cell surface receptors have been used as markers to identify different T_H _subsets. While not exclusive, expression of CXCR3 and CCR5 is associated with T_H_1 cells [1,7,9]; CCR3, CCR4 and CRTh2 with T_H_2 cells [1,7,9]; and CCR4, CCR6 and CD161 with T_H_17 cells [1,14,17,20]. We have previously found that IFN-γ (T_H_1) producing cells are predominantly CD300a/c^+ ^and that both IL-4 (T_H_2) and IL-17a (T_H_17) producing CD4^+ ^T cells are equally distributed between the CD300a/c^+ ^and CD300a/c^- ^subsets [30]. To determine if CD300a/c expression could be utilized to further discriminate among T_H _subsets, we tested for the expression of CD300a/c in T_CM _and T_EM _cytokine producing cells. In these experiments, we classified CD4^+ ^memory cells as those expressing CD45RO and CD27 because these markers remained stably expressed after the *in vitro *stimulation with PMA and ionomycin, whereas the expression of other receptors such as CD62L decreases [48]. Results in Figure 2A show that IFN-γ producing cells in both the T_CM _(CD45RO^+^CD27^+^) and the T_EM _(CD45RO^+^CD27^-^) subsets are mainly CD300a/c^+^. On the other hand, T_CM _cells that produce IL-4 were only slightly enriched in the CD300a/c^+ ^subset, whereas the T_EM _IL-4 producing cells showed no tendency to express or not express CD300a/c. T_EM _cells that produce IL-17a expressed somewhat less CD300a/c, whereas the T_CM _IL-17a producing cells showed no tendency to express or not express CD300a/c. Analysis for IL-2 and TNF-α production demonstrated that T_CM _cells producing these cytokines tend to be CD300a/c^+ ^(Figure 2B).

### CD300a/c is differentially expressed by CD4^+ ^T cells that produce IFN-γ and IL-17a

**Figure 3:**
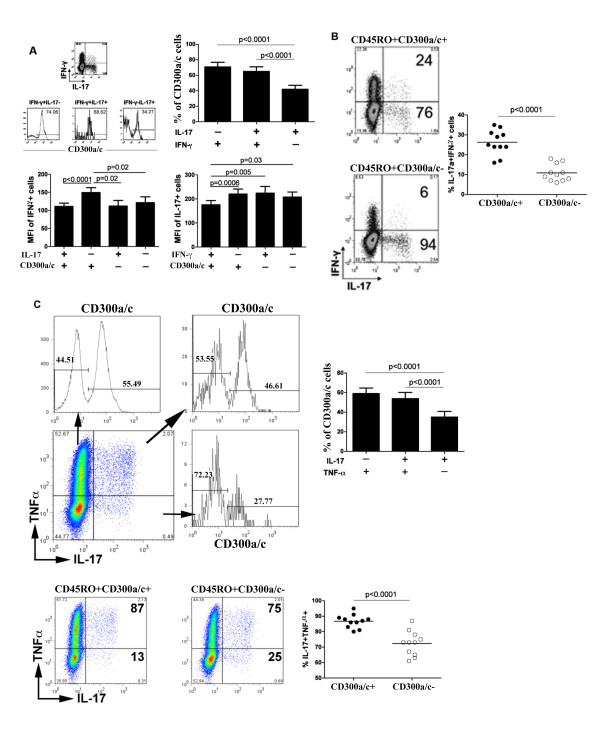
**CD300a/c is differentially expressed on IFN-γ, TNF-α and IL-17a producing CD4^+ ^T cells**. (A) Purified CD4^+ ^T cells were stimulated with PMA and ionomycin for 4-5 h in the presence of GolgiStop (monensin). Then, cells were stained for intracellular production of IFN-γ and IL-17a, and cell surface expression of CD300a/c. Analysis of cells from a representative donor is shown. The bar graphs represent the average ± SEM of the percentage of CD300a/c^+ ^cells within the cytokine producing cells and the average ± SEM of the MFI of IFN-γ expression and IL-17a expression within the cytokine producing cells. Results are from 11 healthy donors. (B) Percentage of cells producing both IFN-γ and IL-17a within the IL-17a producing CD45RO^+^CD300a/c^+ ^and CD45RO^+^CD300a/c^- ^subsets is shown. Analysis of cells from a representative donor (left) and a graph (right) with the results for all donors are presented. (C) The experiments are the same as in A and B, except cells were stained for intracellular production of TNF-α and IL-17a.

Our results show that cells that produce IFN-γ are mostly CD300a/c^+ ^(Figure 2 and [30]), while more IL-17a producing cells are CD300a/c^-^. Several studies have shown that there is a significant portion of cells that produce both cytokines [14,27]. Given the association between IFN-γ production and CD300a/c expression, we reasoned that the production of IFN-γ by some IL-17a producing cells could account for the presence of IL-17a producing cells in the CD300a/c^+ ^subset. In fact, we observed that the IFN-γ^+^IL-17a^+ ^cells are enriched in the CD300a/c^+ ^subset at levels similar to the percentage present for IFN-γ^+^IL-17a^- ^cells, while the IFN-γ^-^IL-17a^+ ^cells tend to be CD300a/c^- ^(Figure 3A). In agreement with our observation of the correlation between CD300a/c expression and IFN-γ production [30], we also observed that cells in the CD300a/c^+^IL-17a^- ^subset produce the largest amounts of IFN-γ and the CD300a/c^+^IFN-γ^+ ^subset produce the lowest levels of IL-17a on a per cell basis, as indicated by the mean fluorescence intensity (MFI) (Figure 3A). The MFI of cytokine staining is a value known to be correlated with the amount of cytokine produced by a T cell [49]. This, together with our previously published results [30], indicates that CD300a/c expression is a marker for cells that produce IFN-γ. Corroboratively, we observed that IFN-γ^+^IL-17a^+ ^cells are predominately CD300a/c^+ ^(Figure 3B). Similar to IFN-γ, TNF-α production is also correlated with CD300a/c expression when both TNF-α and IL-17a production were analyzed and the TNF-α^-^IL-17a^+ ^cells tend to be CD300a/c^- ^(Figure 3C).

**Figure 4:**
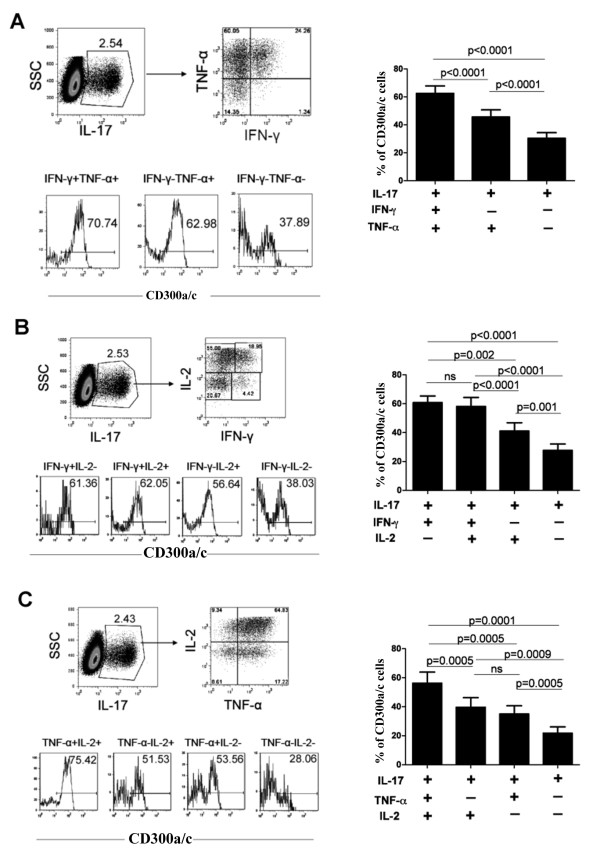
**IL-17a single producing cells tend to be CD300a/c^-^**. Purified CD4^+ ^T cells were stimulated with PMA and ionomycin for 4-5 h in the presence of GolgiStop (monensin). Then, cells were stained for cell surface expression of CD300a/c and intracellular production of IL-17a and other cytokines. A representative donor is shown for (A) IL-17a, for TNF-α and IFN-γ producing cells, (B) IL-17a, IL-2 and IFN-γ producing cells, and for (C) IL-17a, IL-2 and TNF-α producing cells. Graph bars represent the average ± SEM of the percentage of CD300a/c^+ ^cells within the cytokine producing cells. Results are from 8-12 healthy donors.

We further explored the notion that cells producing IL-17a alone tend to be CD300a/c^-^. To do this, we analyzed CD300a/c expression on CD4^+ ^cells producing IL-17a alone or in combination with two other cytokines (Figure 4). We analyzed cells that produce IL-17a, IFN-γ and TNF-α (Figure 4A), IL-17a, IFN-γ and IL-2 (Figure 4B) and cells that produce IL-17a, TNF-α and IL-2 (Figure 4C). We found that cells producing IL-17a alone tend to be CD300a/c^-^, whereas cells producing IL-17a plus one or two additional cytokines tend to be less CD300a/c^-^. More importantly, we found that the IL-17a producing cells that also produce IFN-γ tend to be enriched in the CD300a/c^+ ^subset (Figure 4A and 4B).

### Ligation of CD300a/c with the E59.126 mAb modulates TCR mediated signals on human CD4^+ ^T cells

**Figure 5:**
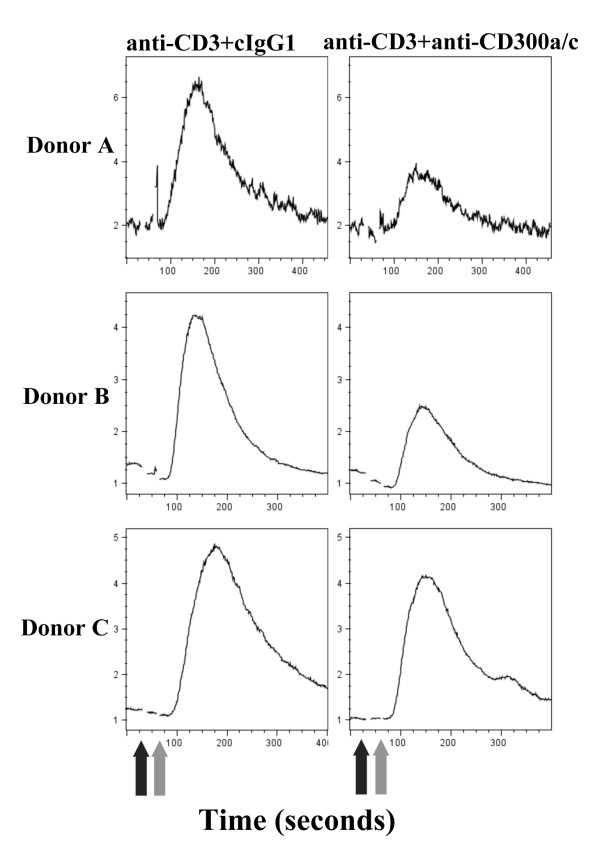
**CD300a/c modulates TCR mediated Ca^2+ ^flux**. CD4^+ ^T cell lines were generated from healthy donors. Cells were loaded with Fluo-4 and Fura-Red. Then, cells were acquired in a flow cytometer, and after a baseline reading of 30 s, anti-CD3 plus anti-CD300a/c mAb or anti-CD3 plus isotype control mAb (black arrows) were added. Goat anti-mouse Ab was added 30 s later to cross-link the primary mAb (grey arrows). Intracellular Ca^2+ ^concentration was measured by the ratio of Fluo-4/Fura Red as a function of time.

Human CD300a and CD300c are two highly homologous receptors expressed on cells of both lymphoid and myeloid lineages with opposing signaling capabilities [31]. Ligation of these receptors with mAb is known to modulate activation signals on a variety of cells, including NK cells, neutrophils, mast cells, B cells and eosinophils [32-34,36,37]. Although several studies have shown that CD300a/c^+ ^and CD300a/c^- ^CD4^+ ^T cells have different features [30,41], to our knowledge there is no report addressing the modulatory role of ligating CD300a and CD300c on TCR mediated signaling. To determine if CD300a/c are capable of regulating human T cell activation signals, we generated CD4^+ ^T cell lines from healthy donors that express high levels of this receptor and measured intracellular Ca^2+ ^flux. Co-ligation of TCR and CD300a/c with mAbs resulted in a significant decrease in intracellular Ca^2+ ^flux, indicating that CD300a/c ligation down-modulates TCR mediated signals (Figure 5). Given that IFN-γ producing cells tend to be CD300a/c^+ ^we asked the question if ligation of CD300a/c with the E59.126 mAb can modulate the production of this cytokine in response to TCR stimulation. To do this, we polarized purified CD4^+ ^T cells under T_H_1 conditions and tested for IFN-γ production. Results shown in Table 1 illustrate that compared with ligation of the TCR alone, co-ligation of the TCR and CD300a/c inhibited TCR mediated production of IFN-γ by CD4^+ ^T cells from three separate donors, but increased the number of IFN-γ^+ ^cells in two donors. These results demonstrate that ligation of CD300a/c with the E59.126 mAb is capable of modulating TCR mediated responses.

**Table 1:** Percentage of IFN-γ producing cells

	cIgG1^1^	Anti-CD300a/c^1^	cIgG1^1^	Anti-CD300a/c^1^
DONORS	Anti-CD3 (0.1 μg)	Anti-CD3 (0.1 μg)	Anti-CD3 (0.5 μg)	Anti-CD3 (0.5 μg)
D1	27	21	42	37
D2	22	23	24	24
D3	11	7	9	9
D4	15	8	31	22
D5	13	17	23	28
D6	35	37	46	55

^1^Isotype control (cIgG1) and anti-CD300a/c mAb are used at a constant concentration of 5 μg per well.

## Discussion

For the past 10-15 years, a remarkable effort has been dedicated to the comprehensive phenotypic and functional characterization of human T cell subsets [50,51]. In addition to the cytokines that CD4^+ ^T cell subsets secrete, they also express a relatively specific pattern of chemokine receptors. For instance, while CCR5 and CXCR3 expression is associated with T_H_1 cells [1,7,9], CCR4 and CCR6 expression is associated with T_H_17 cells [1,14]. In general, the association between the expression of cell surface receptors and intracellular cytokines implies that T cell subsets exhibit differential requirements for effector functions, homing potential, survival and stimulation. However, it should be noted that the broad correlation between cell surface receptor expression and cytokine production patterns is far from perfect [50]. Furthermore, the existence of CD4^+ ^T cells that are able to produce two or more cytokines ascribed to different lineages complicates the classical view of distinct subsets [14,27,29]. Another important question, that recently is under intense investigation, is one of T cell plasticity or flexibility, implying that CD4^+ ^T cell subsets are more appropriately viewed as works in progress rather than terminally differentiated cells [24-26]. For instance, studies in humans have shown the plasticity of T_H_17 cells and their ability to switch into T_H_1 cells, a process that is mediated by IL-12 [24,27,52]. Cell plasticity or flexibility may arise from heterogeneous populations or, alternatively, from flexible genetic programs [25,26].

In addition to chemokine receptors, other cell surface markers have been ascribed to specific CD4^+ ^T cell subsets. For instance, all human T_H_17 cells express CD161 [17,53]. Also, we have previously reported that in peripheral blood the majority of IFN-γ producing cells are CD300a/c^+^, although there was a degree of variability [30]. Nevertheless, the T_H_1 cells that are CD300a/c^+ ^are characterized by their higher production of IFN-γ on a per cell basis and their tendency to be polyfunctional in regard to cytokine production [30]. One of the most groundbreaking classifications of memory T cells was proposed by Sallusto and colleagues. They identified two subsets, T_CM _and T_EM _cells, that are distinguished according to the surface expression of CD45RA and CCR7 [45]. Our results show that both T_EM _and T_CM _CD4^+ ^T cells can be further subdivided into two subsets based in their CD300a/c expression. We have found that T_EM _CD4^+ ^T cells have a higher frequency of CD300a/c^+ ^cells than T_CM _CD4^+ ^T cells. The finding that memory CD4^+ ^T cells can be divided into two populations based on their level of CD300a/c expression raises the possibility of a functional dichotomy related to the level of this expression. In fact we show that CD300a/c^+ ^cells, both in the T_CM _and T_EM _subsets, harbor a higher frequency of IFN-γ producing cells than the CD300a/c^- ^T_EM _and T_CM _CD4^+ ^T cells, which is in agreement with our previous published results showing that T_H_1 cells are mostly, but not entirely CD300a/c^+ ^[30]. Analysis of IL-2 and TNF-α producing cells also show that CD300a/c^+ ^cells show a higher frequency of cytokine producing cells, although this increase is restricted to the T_CM _CD4^+ ^cells. On the other hand, the CD300a/c^- ^subset in the T_EM _CD4^+ ^T cells tends to have a higher number of IL-17a producing cells than the CD300a/c^+ ^cells. In light of this differential CD300a/c expression in the IFN-γ and IL-17a producing cells, we decided to study the expression of this marker on IL-17a producing cells in more detail. Our results show two important findings: first that in *ex vivo *stimulated peripheral blood CD4^+ ^T cells from healthy donors the IFN-γ producing cells are mostly CD300a/c^+^, independently if they produce only IFN-γ or IFN-γ plus IL-17a; and second that, on a per cell basis, the higher IFN-γ producers are CD300a/c^+ ^and do not produce IL-17a at the same time, while the lower IL-17a producers are CD300a/c^+ ^and produce at the same time IFN-γ. This suggests that the IFN-γ and IL-17a higher producers cells are mostly, although not entirely, exclusive and that this correlates with the cell surface expression of CD300a/c. In addition to other factors, this probably reflects the effect of TGF-β1 *in vivo*, as this cytokine has been shown to down-regulate CD300a/c expression and IFN-γ production, while it is important for the generation of T_H_17 cells [30]. Although additional studies are required to further define CD300a/c^+ ^and CD300a/c^- ^T_H_1 and T_H_17 cells, to elucidate for example the degree of plasticity or flexibility of the two T_H_17 cell subsets, our observation that CD300a/c is differentially expressed on these subsets adds another layer of complexity to the growing field of human T_H _cell subsets [1,25,26].

Here we show that cross-linking of CD300a/c and TCR with mAbs is capable of modulating signals evoked by TCR ligation alone. Results presented in Table 1 show that co-ligation of the TCR and CD300a/c inhibited TCR mediated IFN-γ production on polarized T_H_1 cells in three donors while there was an increase in the frequency of IFN-γ^+ ^cells in two donors. These results are reminiscent of those published by Lankry et al [34]. These authors showed that CD300a is indeed an inhibitory receptor able to inhibit human NK cell mediated killing, despite the fact that some NK cell clones were not inhibited in a CD300a dependent manner. They proposed that the absence of the inhibitory signal on those clones was probably due to the presence of CD300c on NK cells that was also recognized and ligated by the same mAbs they used [34]. We believe that this potential ligation of either receptor also may account for the results we have obtained, since we have found that the E59.126 mAb recognizes CD300a and CD300c (data not shown). Both receptors were previously shown to be expressed on human CD4^+ ^T cells, at least at the transcript levels [41]. Furthermore, the recent discovery that CD300c is able to interact and form heterocomplexes with other members of the CD300 family, including CD300a, adds another degree of complexity in the signaling pathways that emanate from these two receptors [43]. Human CD4^+ ^T cells express CD300a and CD300c [31,41] and it may be expected that, depending on the relative expression of each one of these receptors, the signaling outcome after ligation with anti-CD300a/c mAbs, could be different depending on the donors. Clearly, further investigation is required to be done in this regard, and the generation of extensively tested specific mAbs that are able of discriminating both CD300a and CD300c is necessary.

## Conclusions

Taken together, we show that *ex vivo *isolated human T_H_1 and T_H_17 cells differentially express CD300a/c, probably reflecting the different requirements for the *in vivo *generation of both T_H _subsets. Moreover, we show for the first time that ligation of CD300a and CD300c with the E59.126 mAb has an immunomodulatory role on human CD4^+ ^T cells as revealed by its ability to modulate TCR mediated Ca^2+ ^flux and IFN-γ production. Better understanding of the role of the different T_H _subsets based on their CD300a/c expression might be useful for innovative drug development and disease management in the future.

## Methods

### Study population

Buffy coats were obtained from the National Institutes of Health (NIH) blood bank from healthy donors. All study subjects provided written informed consent, in accordance with the Institutional Review Boards of the NIH and the Food and Drug Administration (FDA). FDA specifically approved this study under RIHSC protocol 08-070D.

### Reagents

Antibodies and reagents used in this study were obtained from the following vendors: purified and PE-Cy7 anti-CD3 (clone UCHT1), purified anti-CD28 (clone CD28.2), PE-Cy7 and APC anti-CD4 (clone RPA-T4), APC anti-CD27 (clone 0323), APC anti-CD45RA (clone HI 100), FITC, PE-Cy7 and APC anti-CD45RO (clone UCHL1), APC anti-CD62L (clone Dreg56), FITC and APC anti-IL-2 (clone MQ1-17H12), FITC anti-IL-4 (clone MP4-25D2), FITC anti-IL-17a (clone eBio84DEC17), PE-Cy7 and APC anti-TNF-α (clone Mab11) from eBioscience. PE-Cy7 and Alexa Fluor 647 anti-IFN-γ (clone B27) are from BD Biosciences. Purified and PE anti-CD300a/c (clone E59.126) are from Beckman-Coulter. FITC anti-CCR7 (clone 150503) is from R&D Systems. Goat anti-mouse (GAM) IgG and isotype control IgG1 from Jackson ImmunoResearch Laboratories. Recombinant IL-2 was obtained from the NCI and recombinant IL-12 from R&D Systems. Fluo-4 and Fura-Red were obtained from Invitrogen. PMA and Ionomycin were purchased from Sigma. BD Cytofix/Cytoperm Plus kit with GolgiStop (monensin) was used for intracellular cytokine detection and was purchased from BD Biosciences.

### Cell activation assays and intracellular staining

Human CD4^+ ^T cells were isolated from buffy coats by negative selection with a kit from Miltenyi Biotec (purity was > 93%). For activation experiments, purified CD4^+ ^T cells were cultured in T cell medium: IMDM medium (Invitrogen) containing 10% human AB serum (Valley Biomedical) and supplemented with GlutaMAX (Invitrogen). Cells were stimulated with PMA (50 ng/ml) plus Ionomycin (2 μM) for 4-5 h. GolgiStop (monensin) was added to the cells at the same time than PMA and Ionomycin. Then, cell surface receptor expression and intracellular cytokine staining were determined with the BD Cytofix/Cytoperm Plus kit following the manufacturer instructions. A second set of experiments was performed with purified CD4^+ ^T cells that were polarized under T_H_1 conditions. Cells were cultured in a 12 well plate pre-coated with 1 μg of anti-CD3 and 5 μg anti-CD28 mAb. Four ml of cells at a concentration of 1 × 10^6^/ml were added to the plate and cultured in T cell medium containing 100 U/mL of IL-2 and 10 ng/mL of IL-12. After three days, cells were transferred to non-coated plates adding fresh medium containing cytokines if necessary. At day 8, cells are washed and resuspended in T cell medium containing GolgiStop at a density of 0.5-1 × 10^6^/ml. Two ml of cells were added to each well of a 24 well plate coated with different amounts of anti-CD3, anti-CD300a/c and isotype control IgG1 mAb and incubated for 6-7 h in the presence of GolgiStop (monensin). Then, intracellular IFN-γ was determined with the BD Cytofix/Cytoperm Plus kit. Flow cytometry experiments were performed with a FACS Calibur cytometer (BD Biosciences) and the data were analyzed using the FlowJo software package (Treestar). Lymphocytes were electronically gated based on the side and forward scatter parameters.

### Ca^2+ ^flux assay

Primary human CD4^+ ^T cell lines were obtained by culturing freshly isolated cells in 24 well plates coated with anti-CD3 (1 μg/well) plus anti-CD28 (5 μg/well) mAbs and 100 U/ml of recombinant IL-2. Cells were re-stimulated weekly and used for Ca^2+ ^flux experiments during the third or fourth week. CD4^+ ^T cell lines were washed and resuspended in HBSS (Invitrogen) with 1% FCS at 5 × 10^6 ^cells/ml and labeled with Fluo-4 (2 μg/ml) and Fura Red (5 μg/ml) for 30 min at 30°C. Cells were washed twice and resuspended at 2 × 10^6 ^cells/ml. Prior to activation, cells were incubated at 37°C for 5 min in a water bath, followed by acquisition for 30 s in a FACS Calibur cytometer to establish a baseline. Then, primary mAb were added, and acquisition of cells was continued for 30 s. At this point, primary mAb were cross-linked with goat anti-mouse (GAM) IgG and acquisition was continued for 5-6 more min. Data were analyzed using the FlowJo software package.

### Statistical analysis

Quantitative data were analyzed using GraphPad Prism software. The data were plotted as bar graphs or scatter plots, and pair wise comparisons were examined by two-tailed paired Student's *t*-test with 99% of confidence interval. *P *< 0.05 was considered significant.

## Competing interests

The authors declare that they have no competing interests.

## Authors' contributions

VRS and FB designed the study. VS, JM, QZ and KED performed the experiments, collected and analyzed data. FB and QZ wrote the manuscript. All authors read and approved the final manuscript.
